# Thin Film Growth of a Charge Transfer Cocrystal (DCS/TFPA) for Ambipolar Thin Film Transistors

**DOI:** 10.1021/acsaelm.1c00367

**Published:** 2021-06-02

**Authors:** Wolfgang Rao Bodlos, Sang Kyu Park, Birgit Kunert, Soo Young Park, Roland Resel

**Affiliations:** † Institute of Solid State Physics, Graz University of Technology, Petersgasse 16, 8010 Graz, Austria; ‡ Center for Supramolecular Optoelectronic Materials, Department of Materials Science and Engineering, Seoul National University, 1 Gwanak-ro, Gwanak-gu, Seoul 151-744, South Korea

**Keywords:** organic thin film transistor, ambipolar organic transistor, charge transfer crystal, physical vapor deposition, X-ray diffraction

## Abstract

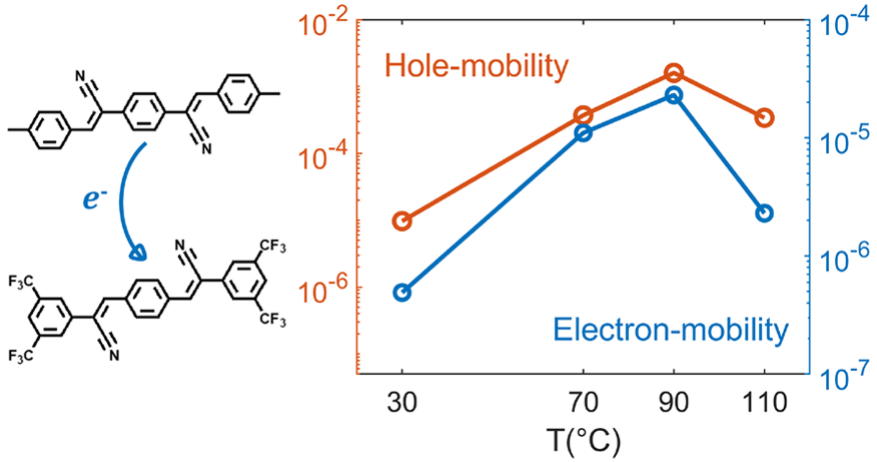

The highly luminescent dicyanodistyrylbenzene-based charge-transfer (CT) cocrystal based on isometric donor and acceptor molecules with a mixing ratio of 2:1 is characterized in the thin film regime. Physical vapor deposited films prepared at different substrate temperatures are analyzed in terms of their thin film structure and transistor performance. The thin film morphologies and crystallographic properties including microstrain and mosaic spread strongly dependent on the substrate temperature. Enhanced crystal growth with rising temperatures leads to a better transistor performance reaching its maximum at 90 °C with a hole and electron mobility of 1.6 × 10^–3^ and 2.3 × 10^–5^ cm^2^ V^–1^ s^–1^, respectively. At higher temperatures performance decreases limited by percolation pathways between the enlarged crystals.

## Introduction

The research on thin film π-conjugated organic semiconductors has made tremendous progress in the last decades and diversified the field in different areas reaching from π-conjugated polymers to oligomers and conjugated small molecule semiconductors.^1,2^ It led to a deep understanding of structure and electrical performance in noncovalently bonded systems and highlights how strongly they define the final performance.^3,4^ In the context of organic field effect transistors one of the most promising approaches uses tailored small molecule semiconductors that self-align during deposition at the surface. These specially designed molecules often show large transfer integrals and small reorganization energies giving them relatively high hole mobilities while maintaining a ease in processability, strong surface crystallization, and simple purification.^2,5−8^ Key challenges however still remain especially concerning performance and stability. A promising approach to tackle these problems uses multicomponent organic molecule blends forming so-called cocrystals, made of two or more distinct chemical species.^9−11^ They bind noncovalently by heteromolecular interactions, such as halogen or hydrogen bonds, π–π interactions, or charge transfer interaction. Especially the latter has widely been used to form stable, densely packed organic cocrystals consisting of stacked donor (D) and acceptor (A) molecules.^12−14^ Quantum chemical calculations suggest that the molecular orbital interaction of such complexes allows efficient electronic coupling of holes and electrons via superexchange making them ideal for ambipolar transistor devices.^15−18^ In addition D–A complexes have already achieved a considerable performance in the context of optoelectronic applications,^13,19−21^ albeit these bottom-up grown crystals lack in practical applicability. Very recently this issue was addressed by designing and synthesizing charge-transfer cocrystals with isometrically structured dicyano-distyrylbenzene-based D and A molecules showing high charge-transfer electro- luminescence in a single active-layered organic light-emitting transistor (OLET). The films were grown by physical vapor deposition (PVD) ensuring both large area processability and reasonable performance. Some questions however were left unanswered: is the same CT cocrystal structure present in the thin film regime? How do the morphologies and crystalline properties, both fundamental parameters regulating optoelectronic performance, evolve with temperature? Here we are going to answer these questions by in-depth topographic and crystallographic studies as well as device characterization. The results not only provide mechanisms of thin film growth but also engender structure–morphology–property correlations of thin film transistor devices.

## Experimental Section

**Figure 1:**
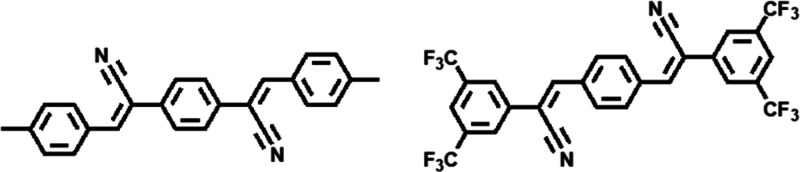
Chemical structure of the 2M-aDCS donor molecule (left) and the CN-TFPA acceptor molecule (right).

The charge-transfer complex is created by combining the donor 1,4-bis(1-cyano-2-phenylethenyl)benzene (3) (DCS) with the acceptor ((2*Z*,2′*Z*)-3,3′-(1,4-phenylene)bis(2-(3,5-bis(trifluoromethyl)-phenyl)acrylonitrile) (TFPA). Both molecules were synthesized by the Knoevenagel condensation reaction.^12,22^ The DCS and TFPA molecules are shown in Figure 1.

The polycrystalline powder of the charge-transfer complex was obtained by recrystallization using toluene as a solvent. The stoichiometric donor to acceptor ratio was chosen to be 2:1.^23^

**Figure 2:**
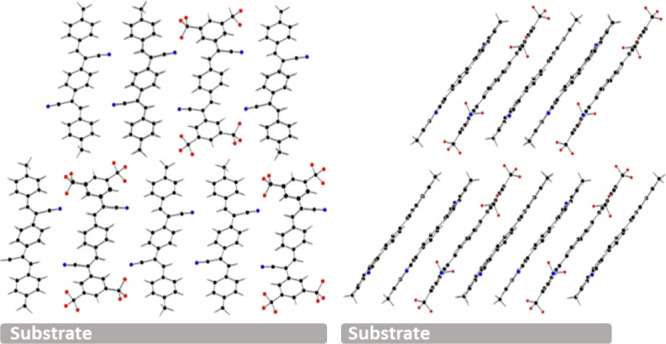
Front and side view of the molecular packing of bulk DCS/TFPA single crystalline films.

This mixing ratio is reflected in the molecular arrangement within the bulk crystal structure solution of single crystals shown in Figure 2.

The thin films of the complex were prepared by physical vapor deposition (PVD) from the mixed powder. As a substrate, 1 cm × 1 cm Si wafers with a 300 nm thermal oxide layer were used. The substrates were pretreated with an octadecyltrichlorosilane (OTS) self-assembling monolayer to increase the hydrophobicity.^24,25^

The powder was evaporated in a vacuum chamber under a low base pressure of 4 × 10^–6^ Torr. The deposition rate was continuously monitored by a quartz crystal microbalance located near to the substrate, keeping the rate constant between 0.3 and 0.4 Å /s. The deposition was stopped at a nominal film thickness of 400 Å.

The films were characterized by three different X-ray based methods: specular X-ray diffraction (XRD), X-ray reflectivity (XRR), and grazing incidence X-ray diffraction (GIXD). The specular X-ray scattering studies (XRR and XRD) were performed on a PANalytical Empyrean system using K_α_ radiation from a sealed Cu-tube (λ = 1.54178 Å). At the primary side, the radiation passes through a multilayer X-ray mirror generating a monochromatic and parallel beam with a height of 100 μm; at the secondary side, a 100 μm receiving slit and a 0.02 rad Soller slit were used in combination with a PANalytical PIXcel 3D detector operating in the 0D point (XRR) or 1D line (XRD) mode. The data are plotted as a function of the out-of-plane component of the scattering vector *q*_*z*_ via *q*_*z*_ = sin θ, λ being the wavelength and θ being half of the scattering angle 2θ.

The peak width analysis was performed by using the full width at half-maximum of the Bragg peaks.^26^ Rocking curves were measured in coplanar geometry using the specular 003 Bragg peak.

Grazing incidence X-ray diffraction (GIXD) measurements were performed at the KMC-2 beamline at BESSY II (Berlin, Germany). An X-ray wavelength of 1.00 Å was used with an incident angle of α_i_ = 0.13° to enhance the scattered intensities of the adsorbate. The data were collected on a 2D cross-wire detector (Bruker). The diffraction pattern was transformed into reciprocal space by using the software *xrayutilities*.^26^ The calculation of the peak positions and structure factors was performed using the custom-made software PyGID.^27^

Fluorescence optical microscopy images were performed on a Leica DM LP optical microscope equipped with a Leica DFC420 C camera by illuminating UV light using an excitation filter.

The atomic force microscopy (AFM) measurements were performed on a Bruker Nanoscope III multimode SPM modular scanning probe system. It was operated in the tapping mode using a RTESP cantilever.

**Figure 3:**
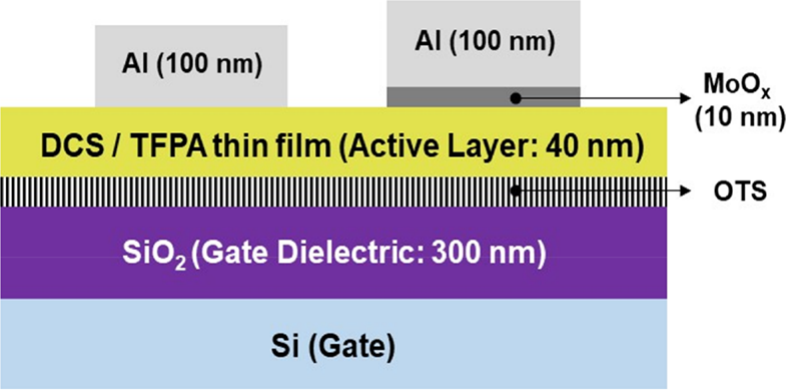
Schematic illustration of the thin film transistor layout.

The charge carrier mobility of the prepared films was evaluated by fabricating top-contact field-effect transistors with the layout illustrated in Figure 3. The D–A complex active layer was deposited onto OTS treated SiO_2_/Si substrates with a 300 nm thick SiO_2_ layer serving as the gate dielectric and the Si layer acting as the gate electrode. Above the active layer, asymmetric electrodes were deposited under a high vacuum of 1.3 × 10^–6^ Torr. One of the asymmetric electrodes is based on MoOx (100 Å)/Al (1000 Å) and the other on Al (1000 Å). Considering the previously reported highest occupied molecular orbital (HOMO: −5.7 eV) and the lowest unoccupied molecular orbital (LUMO: −3.7 eV) of the D–A complex the MoOx/Al electrode facilitates hole injection and the Al electrode electron injection.^23^

The mobility (μ) values were determined by
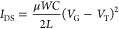
where *I*_DS_ is the drain–source current, *V*_G_ is the gate voltage, *V*_T_ is the threshold voltage, *W* is the channel width of the devices (30–50 μm, measured by optical microscope), *L* is the channel length of the devices (ca. 1000 μm, measured by optical microscope), and C is the capacitance of the 300 nm thick SiO_2_ dielectric layer (11 nF cm^–2^). μ_avg_ describes the average value over 10 samples while μ_max_ indicates the best out of each series of measurements. The measurements were performed within a N_2_-filled glovebox using a Keithley 4200 SCS to avoid potential trap state formations in air. During the electrical measurement, the devices were kept in the dark to avoid possible photoinduced cis–trans isomerization of dicyanodistyrlbenzene derivatives (donor and acceptor) and photoinduced carrier generation.

## Results

**Figure 4:**
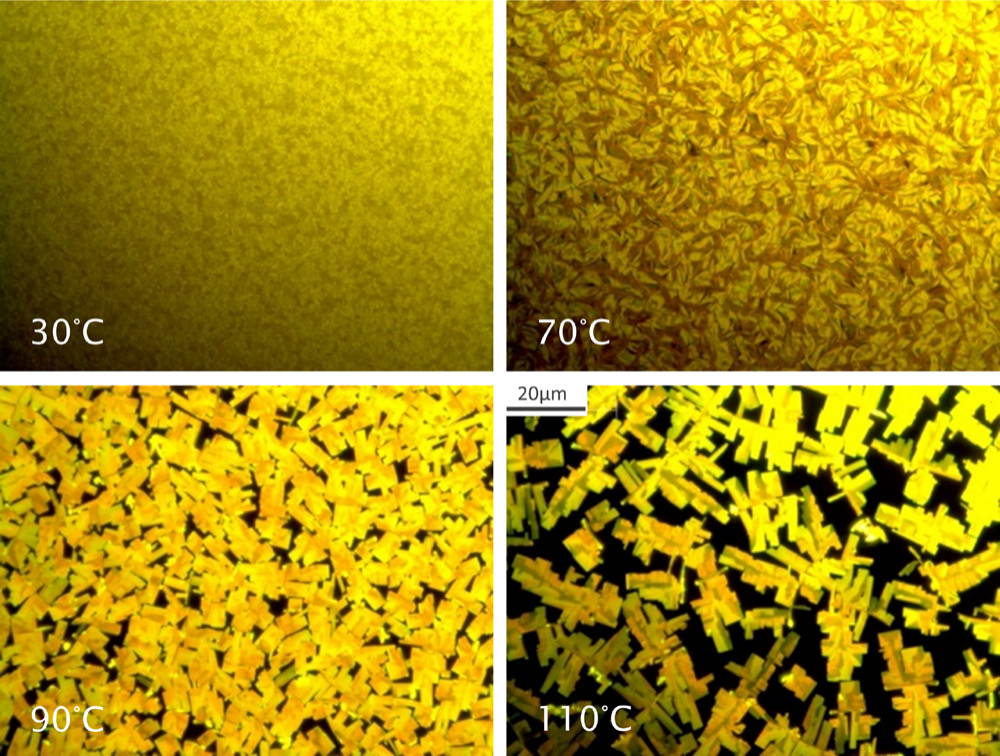
Optical microcopy images of DCS/TFPA thin films deposited at different substrate temperatures onto OTS treated silicon oxide surfaces.

The optical microscope images in Figure 4 give a picture of the thin film structure. Different substrate temperatures during deposition lead to significantly different surface morphologies. At low temperatures a mound like morphology is observable while toward higher temperatures micrometer big islands with planar faces and rectangular shapes appear. At low temperatures the domain size is smaller but the surface is completely covered. As the temperature increases the domain size increases while the surface coverage decreases. From optical microscopy a 2D like crystal growth is visible at low temperatures that goes over into a 3D growth at elevated temperatures.

**Figure 5:**
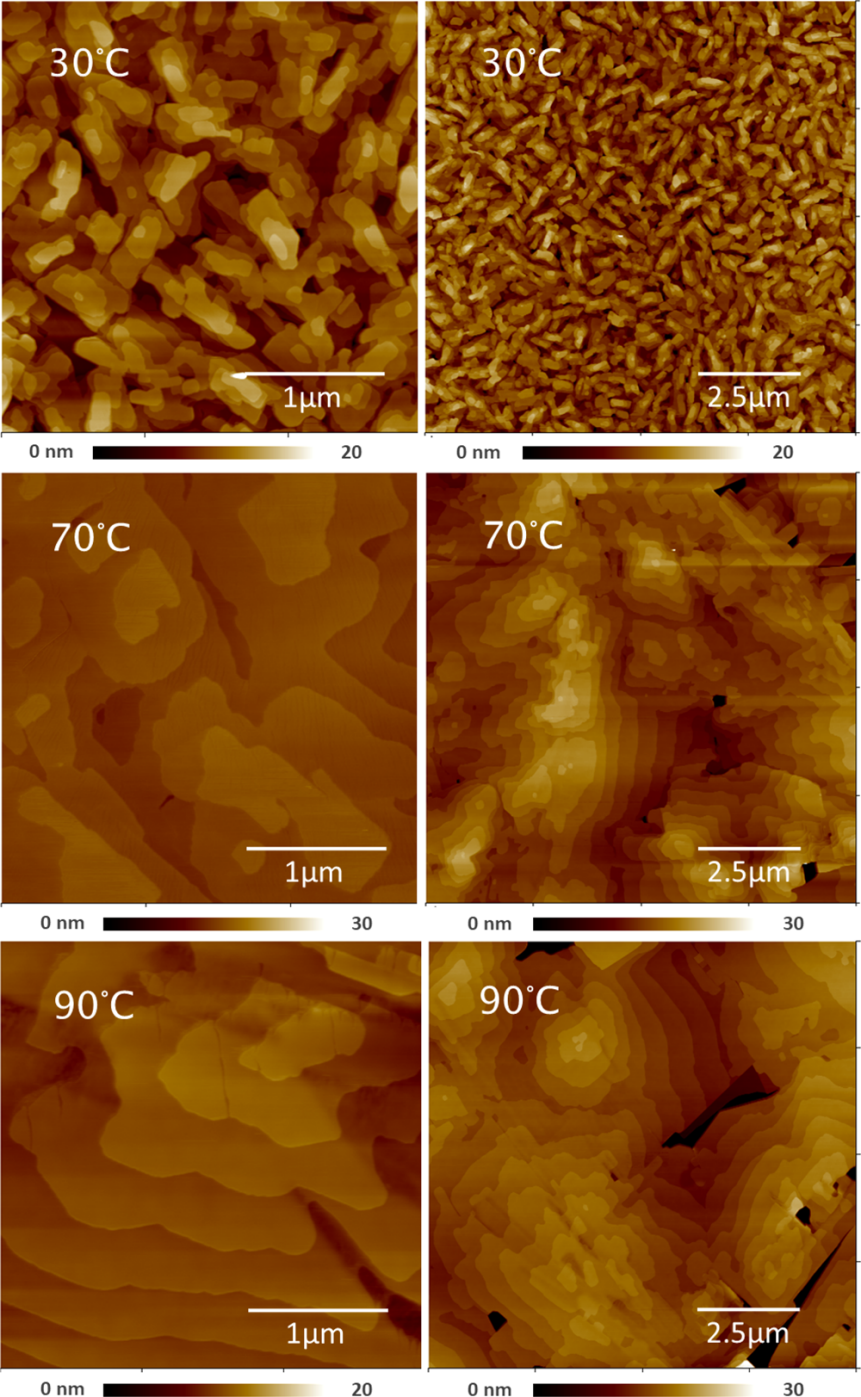
Atomic force microscopy images of DCS/TFPA thin films deposited at different substrate temperatures onto OTS treated silicon oxide surfaces: 3 μm × 3 μm (left) and 10 μm × 10 μm (right).

AFM measurements confirm the observations from the optical microscope images in a higher resolution range. They are shown in Figure 5. At low temperatures a significantly higher number of islands is visible. They form a mound rich surface that covers the underlying substrate nearly fully. Toward higher deposition temperatures enhanced terrace like structures are visible hinting toward an improved vertical and horizontal crystal growth. The underlying substrate starts to become apparent in the 10 μm × 10 μm images at 70 °C suggesting an onset of reduced surface coverage. At 90 °C the trend continues; the terraces gain in size and the features become even larger. The uncovered surface areas gain in size exposing the underlying substrate and reducing the coverage further.

**Figure 6:**
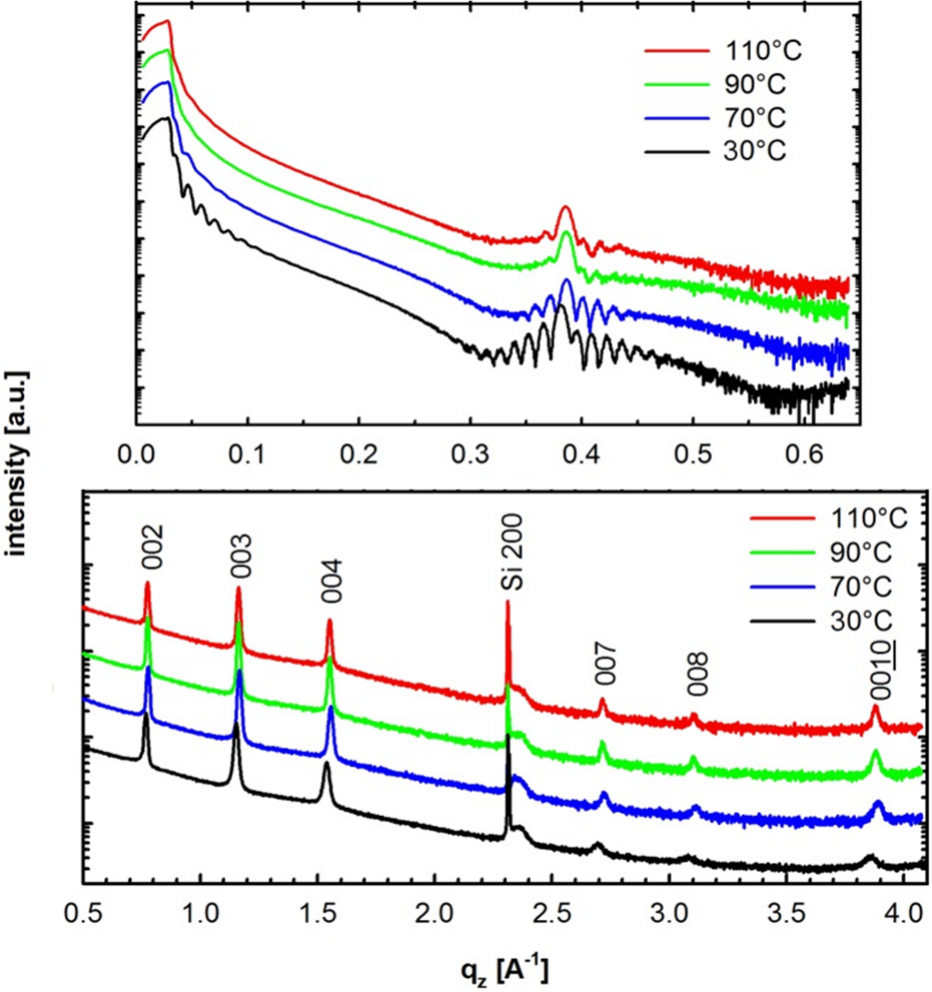
X-ray reflectivity (top) and X-ray diffraction (bottom) measurements of DCS/TFPA thin films deposited at different substrate temperatures onto OTS treated silicon oxide surfaces.

The thin film structure and crystalline packing were analyzed in detail using different X-ray based methods. XRR and XRD measurements are shown in Figure 6 complementing each other in the measurement range. The XRR measurements (top) contain detailed information about the thin film morphology. The oscillations visible between *q*_*z*_ = 0.03 Å^–1^ and *q*_*z*_ = 0.15 Å^–1^ are XRR-Kissing fringes that can be related to the homogeneity of the deposited films and give an estimation of the thickness and roughness. They are strongly pronounced at 30 °C but lose their intensity toward higher temperatures. It reveals the presence of a defined layer at 30 °C which was fitted through a double layer model consisting of an OTS monolayer (23 Å) and a 436 Å thick deposited film with a density of 1.36 g cm^–3^ and a roughness of 35 Å. This model is very reasonable when considering the nominal deposition thickness of 400 Å and the ideal density of 1.396 g cm^–3^ for the DCS/TFPA complex. For higher temperatures the XRR measures indicate that the films get rougher and more inhomogeneous toward 110 °C. It fits to the crystal growth behavior with increasing temperature visible in Figures 4 and 5.

The second notable feature in the XRR measurements is the Bragg peak at *q* = 0.38 A^–1^ with its Laue oscillations to the left and to the right of the peak indicating a crystallite size of around 400 Å which is in the regime of the layer thickness. Based on the position and intensity ratio of the peak it corresponds to the 001 peak of the bulk crystal structure.^23^Figure 6 (bottom) shows the higher orders of the 001 Bragg peak up to the 0010 order measured by XRD. The intensity ratios with the diminishing 005 and 009 peak match to the known crystal structure.^23^ At higher preparation temperatures the peaks are narrower and of slightly higher intensity.

**Figure 7:**
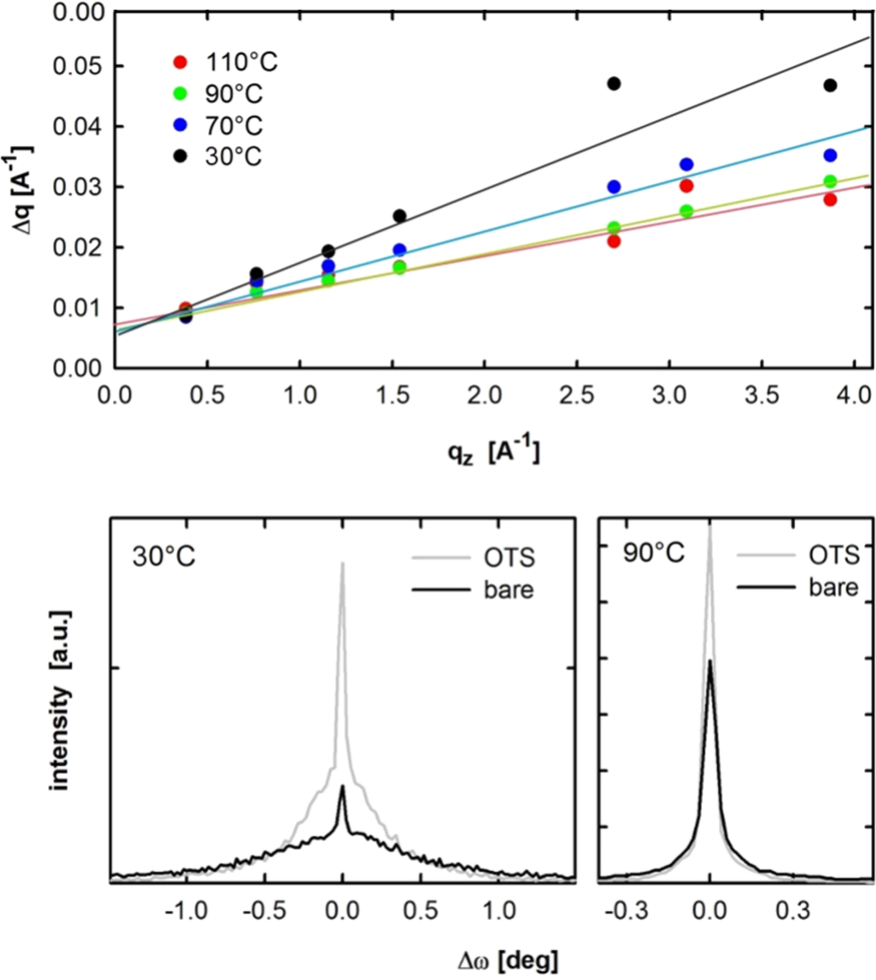
Williamson–Hall analysis (top) on DCS/TFPA thin films deposited at different substrate temperatures onto OTS treated silicon oxide surfaces. Rocking curve measurements (bottom) on films prepared onto OTS treated and bare silicon substrates at room temperature and 90 °C.

The measured XRD peaks were further investigated by performing a Williamson–Hall analysis shown in Figure 7 (top). The method analyzes the peak width of the Bragg peaks (Δ*q*) as a function of their peak position (*q*) and was performed on the 001 and its higher order reflections. By using linear regression and evaluating the slope of the straight line, the micro strain was determined. It gives a measure for the plane distance variation in relation to the plane distance itself () and is caused by defects.^28,29^ A decrease in slope with increasing temperature is observed: 0.012 at 30 °C, 0.008 at 70 °C, 0.006 at 90 °C, and 0.005 at 110 °C.

Rocking curve measurements on the 003 peak shown in Figure 7 (bottom) give an estimation of the mosaic spread i.e. misorientation of the 001-oriented crystals at the surface. At 90 °C a peak width of 0.07° is measured regardless if prepared with or without an OTS layer underneath. The peak is sharper in the presence of the OTS layer indicating a better alignment of the crystals in the specular direction. At 30 °C a broad and a narrow peak overlap. The broad base has a peak width of 0.21° (OTS) and 0.33° (bare) while the sharp peak on top shows a peak width of 0.09° and 0.11°. Also here a better crystal alignment is suggested with the OTS layer. Comparing 30 °C with 90 °C the overall peak width decreases significantly with increasing temperature. The rocking curve measurements therefore suggest that the mosaic spread decreases with increasing substrate temperature.

**Figure 8:**
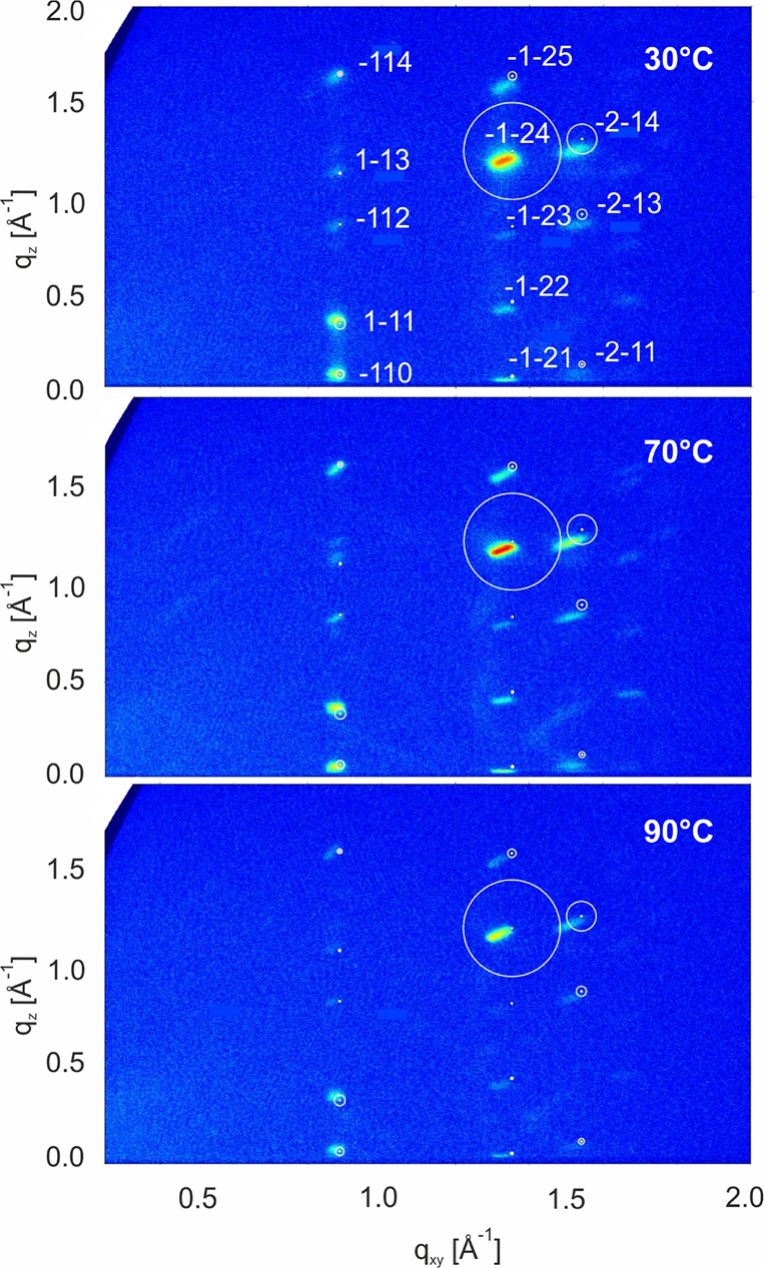
Grazing incidence X-ray diffraction pattern of DCS/TFPA thin films deposited at 30, 70, and 90 °C onto OTS treated silicon oxide surfaces. White markers represent the calculated diffraction pattern with the dots indicating the peak positions and the area of the white circles surrounding them representing the peak intensity.

In a next step the thin films were investigated by grazing incidence X-ray diffraction measurements. The corresponding GIXD patterns are shown in Figure 8. The numerous visible peaks are in accordance with a 001 orientation of the bulk single crystal phase parallel to the surface. The positions as well as the intensities match well for the in total 13 observed diffraction peaks. For the films prepared at 110 °C the enhanced crystallite size leads to a statistical problem in which not all orientations are present within the beam making the data set incomplete.

The crystal structure analysis by XRR, XRD, and GIXD all show the bulk phase of the DCS/TFPA complex discussed in a previous study to be present in the thin film regime.^23^ In this structure the molecules orient toward the surface as illustrated in Figure 2. The molecules alternate with a 2:1 mixing ratio packing closely and partially interlocking with the side chains. They take an inclination angle of ∼54° toward the surface. The π–π orbital stacking distance of the aromatic cores is 3.5 Å.

**Figure 9:**
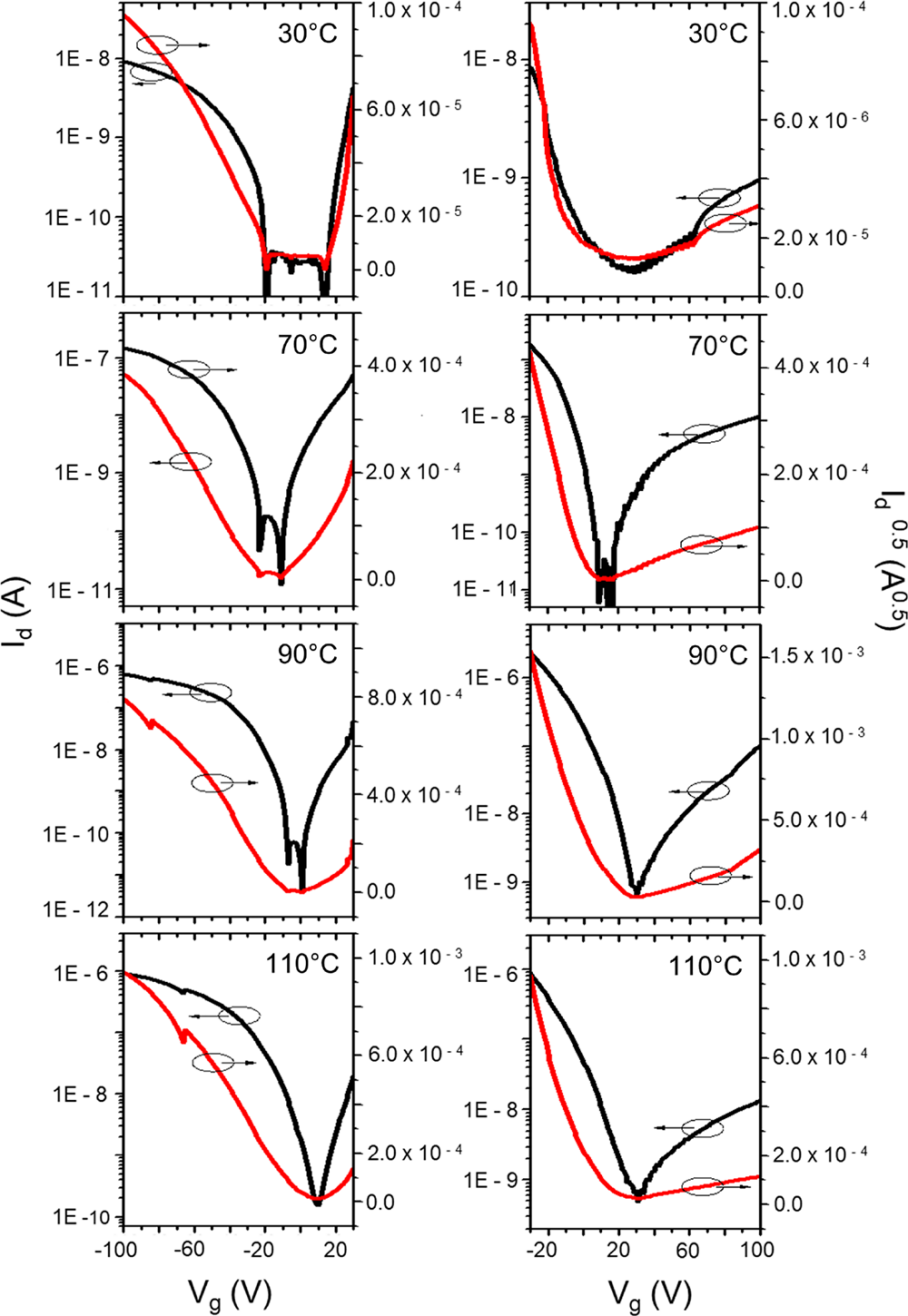
Charge transport mobility measurements demonstrating the *I*–*V* characteristics of DCS/TFPA thin films deposited at different substrate temperatures onto ODTS treated silicon oxide substrates.

**Figure 10:**
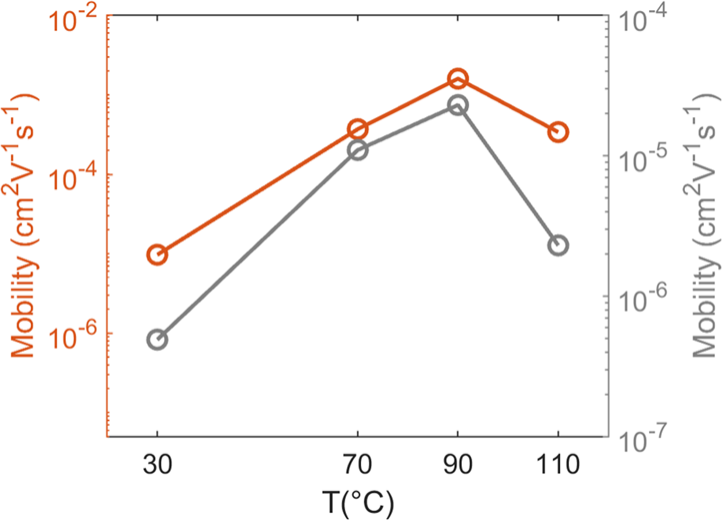
Average electron (red) and hole (gray) mobility measurements of DCS/TFPA thin films deposited at different substrate temperatures onto OTS treated silicon oxide surfaces.

The charge carrier mobility of the films deposited at different substrate temperatures was evaluated in thin film transistors. The exact transfer characteristics are given in Figure 9 with the red curves representing the source drain voltage (*I*_d_), and the black curves the square root of it. The calculated mobility values from these curves are shown in Table 1 together with the on/off ratio and the threshold voltage. These measurements reveal an ambipolar transistor behavior. Figure 10 illustrates how the charge carrier mobility initially increases with the substrate temperature. It reaches a maximum at 90 °C with 2.4 × 10^–3^ cm^2^ V^–1^ s^–1^ in the p-channel regime and 2.3 × 10^–5^ in the n-type regime. At 110 °C both mobility values decrease again.

**Table 1:** Charge Transport Mobilities (μ_avg_ and μ_max_) Together with the On/Off Ratio (*I*_on_/*I*_off_) and the Threshold Voltage (*V*_th_) for DCS/TFPA Thin Films Prepared at Different Substrate Temperatures Grown on OTS Treated Silicon Substrates

deg Celsius	μ_avg_ (μ_max_) [cm^2^ V^–1^ s^–1^]	*I*_on_/*I*_off_	*V*_th_ [V]
p-channel			
30	9.7 × 10^–6^ (1.9 × 10^–5^)	10^1^–10^2^	–4
70	3.7 × 10^–4^ (9.2 × 10^–4^)	10^3^–10^4^	–12
90	1.6 × 10^–3^ (2.4 × 10^–3^)	10^3^–10^4^	–14
110	3.6 × 10^–4^ (9.9 × 10^–4^)	10^2^–10^3^	–4
n-channel			
30	4.9 × 10^–7^ (1.3 × 10^–6^)	10^0^–10^1^	–26
70	1.1 × 10^–5^ (3.2 × 10^–5^)	10^1^–10^2^	11
90	2.3 × 10^–5^ (7.5 × 10^–5^)	10^1^–10^2^	10
110	2.3 × 10^–6^ (1.0 × 10^–5^)	10^0^–10^1^	32

## Discussion

This study elaborates how CT cocrystalline thin films can be grown by PVD and demonstrates the influence of substrate temperature during deposition. At all temperatures it forms the same bulk crystal structure but with a significantly different thin film morphology. At low temperatures defined layers of high surface coverage are observed in the optical microscope, AFM and by XRR. With increasing temperature, a rapid roughening of the thin film surface occurs with the crystallites growing bigger and becoming visible in the optical microscope and AFM as well as showing more intense and narrower XRD Bragg peaks. The surface coverage however decreases leading to a partially uncovered surface at 110 °C.

The observations concerning the layer coverage and the crystal size complement each other when understanding it as a result of rather strong 2D layer growth at low temperatures that goes over into a 3D growth through enhanced diffusion at elevated temperatures.^30,31^ The enhanced 3D growth can explain the reduced visible coverage.

The Williamson–Hall and rocking curve measurements indicate less microstrain, mosaic spread, and misorientation at higher temperatures. The crystal quality improves with an increased size and less defects. That favors more structural order toward the surface and within the crystals.^32,33^

**Figure 11:**
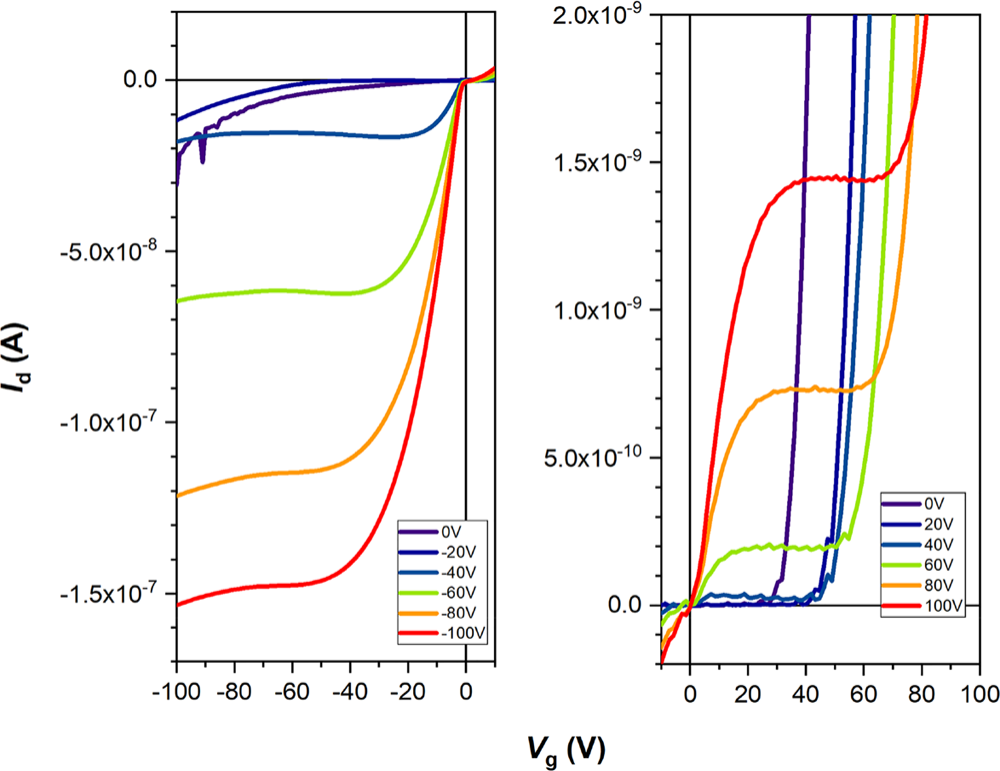
Representative output characteristics measured in hole and electron enhancement modes, using a thin-film device prepared at 90 °C.

The substrate temperature dependent transistor performance is well elucidated by the above observations. Charge carrier mobility measurements on the films prepared at different temperatures reveal an ambipolar behavior showing electron as well as hole mobility. In contrast to the single-crystal transistors based on the same D–A pair, the thin film devices perform significantly better during hole than electron transport.^23^ This imbalance in ambipolarity can be attributed to the presence of grain boundaries weighing the trapping of electrons stronger than contact issues,^34^ especially given the fact that *I*_d_ increases linearly in the small *V*_d_ regime of the output characteristics shown in Figure 11.

Depending on the preparation temperature the mobility appears to increase until 90 °C and then decrease. This interpretation agrees with the morphological observations that show a steady increase of the lateral crystallite size with increasing temperature and with a decrease in the number of grain boundaries. Above 90 °C however, the drop in mobility is explainable with the decrease in surface coverage which reduces the amount of possible transporting pathways. The charge carrier transport becomes describable by percolation theory that predicts a percolation threshold with further increase of substrate temperature.^35,36^ This detailed study of charge carrier transport in relation to the thin film morphology highlights how important crystalline order is to achieve high performance organic thin film transistors.

## Conclusion

Thin film studies on the DCS/TFPA complex show a strong influence of the deposition temperature on the structure, morphology and electrical performance of the PVD prepared films. The crystallite size increases with the substrate temperature significantly while the coverage starts to decrease around 90 °C.

This has direct influence on the ambipolar transistor behavior of the molecule showing an increasing charge carrier mobility toward 90 °C, reaching a maximum of 2.4 × 10^–3^ cm^2^ V^–1^ s^–1^, and then decreasing toward 110 °C becoming limited by percolation pathways between the individual crystals.
